# Towards Non-Invasive Methods in Measuring Fish Welfare: The Measurement of Cortisol Concentrations in Fish Skin Mucus as a Biomarker of Habitat Quality

**DOI:** 10.3390/ani9110939

**Published:** 2019-11-08

**Authors:** Annaïs Carbajal, Patricia Soler, Oriol Tallo-Parra, Marina Isasa, Carlos Echevarria, Manel Lopez-Bejar, Dolors Vinyoles

**Affiliations:** 1 Department of Animal Health and Anatomy, Veterinary Faculty, Universitat Autònoma de Barcelona, Bellaterra, 08193 Barcelona, Spain; manel.lopez.bejar@uab.cat; 2 Department of Evolutionary Biology, Ecology and Environmental Sciences, Universitat de Barcelona, Avinguda Diagonal 643, 08028 Barcelona, Spain; patrisoler89@gmail.com (P.S.); d.vinyoles@ub.edu (D.V.); 3 Department of Animal and Food Science, Veterinary Faculty, Universitat Autònoma de Barcelona, Bellaterra, 08193 Barcelona, Spain; oriol.tallo@uab.cat; 4 Cetaqua, Centro tecnológico del agua, Cornellà de Llobregat, 08940 Barcelona, Spain; marina.isasa@cetaqua.com (M.I.); cechevarriadc@cetaqua.com (C.E.)

**Keywords:** non-invasive, bioindicator, pollution, stress, welfare, constructed wetland, glucocorticoid, urban river

## Abstract

**Simple Summary:**

The analysis of circulating cortisol has been by far the most common method used as a means to assess fish stress responses and, thus, animal welfare. To avoid many of the drawbacks inherent to blood sampling, cortisol can be less-invasively detected in fish skin mucus. The measurement of cortisol in skin mucus however, has, to date, only been demonstrated as suitable for farm fish, although its application to free-ranging animals would offer many advantages. The present study was therefore designed to evaluate the applicability of skin mucus cortisol analysis as a potential tool to assess habitat quality. To that end, wild fish residing in environments of different habitat quality were sampled for blood and skin mucus. First, several physiological endpoints typically used as indicators of exposure to pollutants were accurately related to the habitat quality in the Catalan chub (*Squalius laietanus*). Second, cortisol levels in blood were also compared between habitats, and they were successfully correlated to skin mucus cortisol concentrations. Finally, we contrasted the patterns of response of all the endpoints assessed to skin mucus cortisol levels across the sites. The strong linkages detected in this study provide new evidence that the measurement of cortisol in skin mucus could be potentially used as a biomarker of habitat quality in freshwater fish.

**Abstract:**

Cortisol levels in fish skin mucus have shown to be good stress indicators in farm fish exposed to different stressors. Its applicability in free-ranging animals subject to long-term environmental stressors though remains to be explored. The present study was therefore designed to examine whether skin mucus cortisol levels from a wild freshwater fish (Catalan chub, *Squalius laietanus*) are affected by the habitat quality. Several well-established hematological parameters and cortisol concentrations were measured in blood and compared to variations in skin mucus cortisol values across three habitats with different pollution gradient. Fluctuations of cortisol in skin mucus varied across the streams of differing habitat quality, following a similar pattern of response to that detected by the assessment of cortisol levels in blood and the hematological parameters. Furthermore, there was a close relationship between cortisol concentrations in skin mucus and several of the erythrocytic alterations and the relative proportion of neutrophils to lymphocytes. Taken together, results of this study provide the first evidence that skin mucus cortisol levels could be influenced by habitat quality. Although results should be interpreted with caution, because a small sample size was collected in one studied habitat, the measurement of cortisol in skin mucus could be potentially used as a biomarker in freshwater fish.

## 1. Introduction

Throughout their lifetime, wild fish face many challenges of the aquatic environment that can impose considerable stress and reduce their welfare [1]. These challenges can be either natural or have an anthropogenic origin, and, depending on the magnitude and duration, they can cause acute or chronic stress responses [2,3]. Acute stress responses, such as those triggered by a predator attack or certain unpredictable weather conditions, can facilitate survival [4], whereas long-term stressors, like exposure to environmental pollution, are associated with a wide range of maladaptive effects [5] that may, ultimately, lead to loss of biodiversity [6,7]. Accordingly, understanding the causes and effects of environmental disturbances on fish physiology may help developing conservation strategies to enhance restoration and protect freshwater ecosystems [5,8].

An economical and practical option that can give a substantial amount of information about the overall health status of individuals is the peripheral blood test [9,10]. The analysis of red blood cells (RBCs) allows the detection of DNA damage and alterations by the assessment of erythrocytic nuclear abnormalities (ENA), circulating micronuclei (MN), and senescent (SE) and immature (IE) erythrocytes [11,12]. The detection of RBC abnormalities has actually been widely used as an indicator of exposure to genotoxic and mutagenic contaminants [13,14,15]. In parallel, relative white blood cell (WBC) count can be obtained, which offers a very common measure of stress and innate immune response [16]. In particular, the relative proportion of neutrophils to lymphocytes has been successfully applied as a measure of prolonged pollutant exposure [9,17,18]. Other uses of blood samples in ecotoxicology include the quantification of glucocorticoid (GC) hormones, such as cortisol, to assess the stress response [19,20]. Cortisol is the main GC in teleost fish secreted after the activation of the hypothalamic–pituitary–interrenal (HPI) axis in response to acute and chronic stress [2,21]. Analyses of cortisol levels in blood and, more recently, in whole-body and the surrounding water have been effectively used to monitor environmental stress responses [22,23,24]. Blood, whole-body, and the surrounding water sampling, however, present clear limitations when being applied in wild population studies. First, blood collection is an invasive technique that the process by itself may provoke further stress and thus it can potentially compromise the animal’s welfare. Similarly, whole-body cortisol analysis involves sacrifice of the specimens [22]. And finally, collection of the holding water requires fish restriction in a bucket, which can cause additional stress. Moreover, this technique is difficult to apply in the wild [24]. Fish scales can also accumulate cortisol [25,26]; however, their potential as biomarkers of habitat quality deserves further investigation [27]. Cortisol analysis in fish skin mucus has recently gained considerable attraction, especially because the sampling method is much less invasive compared to the aforementioned techniques [28,29,30]. Skin mucus cortisol levels have been shown to reflect circulating concentrations in several species of farm fish [26,30,31,32], but there is yet no evidence of such a relationship in free-ranging species. In addition, this method has, to date, only been applied in strictly controlled environments [30,33], hence its applicability in uncontrolled, natural environments remains to be explored. Therefore, the present study aimed to examine whether skin mucus cortisol concentrations (MCC) from the freshwater fish Catalan chub, *Squalius laietanus* [34], are affected by the habitat quality to further develop non-invasive biomarkers in free-living fish. Catalan chub was chosen, as this species has demonstrated to be a good candidate for freshwater biomonitoring using blood tests [11]. It is well known that understanding changes in cortisol levels is not a simple process, especially when measuring cortisol in wild animals by using alternative samples other than blood [6,9]. Given that the measurement of cortisol in skin mucus is a novel method, other physiological endpoints of the effects of pollution in fish were assessed to better interpret cortisol fluctuations in this matrix. Several hematological parameters (RBC anomalies and altered WBC counts) were measured in parallel, since, as previously mentioned, they have successfully been used as indicators of health condition in the Catalan chub [11], as well as in many other species (reviewed above). 

This study was carried out in a populated and industrialized urban river, where efforts are being made to minimize the environmental impacts and recover the aquatic fauna throughout the performance of constructed wetlands. Catalan chub were sampled from a non-impacted upstream site and two downstream polluted sites, located within the constructed wetland system, in order to compare fish residing environments of different habitat quality. Initially designed specifically for wastewater treatment, constructed wetlands are nowadays an important component of urban ecosystems since they play a crucial role in environmental pollution control [35,36,37]. Constructed wetlands are macrophyte-based systems that remove pollutants through a combination of physical, chemical, and biological processes [36,38]. Wetlands’ performances, though, need to be periodically monitored [39]. The described methodologies for wetland monitoring include physical and chemical techniques that provide information about the amount of pollutants present in the water. Nevertheless, these tools do not give insight into how living organisms cope with water contaminants [39]. On this basis, the present study was carried out in a constructed wetland system to highlight the need to apply techniques that provide information about how animals perceive and adapt to their environment.

## 2. Materials and Methods 

### 2.1. Study Area and Field Sampling 

**Figure 1:**
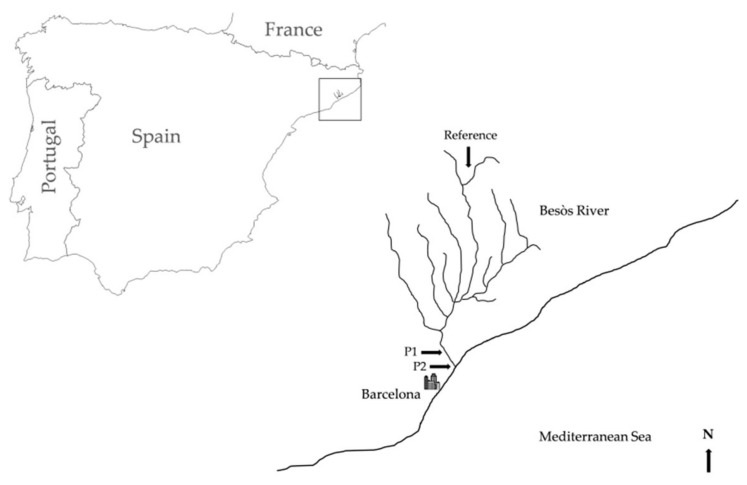
Map showing the location of the three sites sampled in the present study (Reference, P1 and P2) within the Besòs River, in north-east Spain.

In order to study the influence of the habitat quality on skin mucus cortisol concentrations, individuals were sampled from two sites within a wetland system (Besòs River Park, NE Spain), each of which represents a different stage of biodegradation of water pollutants (P1 and P2), and a reference non-impacted upstream site located outside the wetlands (Figure 1). The reference site was set in a small tributary (Riera d’Avencó), 49.6 km distant from the site P1. The sampling site P1 was placed at the beginning of the constructed wetland, 2.9 km distant from P2, which was located at the end of the overall wetland system and 3.6 km to the river mouth. The Besòs River is an urban river adjacent to the City of Barcelona (Catalonia, NE Spain). During the 1970s and 1980s this river was declared the most polluted river in Europe. Fish populations are slowly recovering, making it easy to find an abundance of differences between nearby sites. 

Sampling areas were determined following the protocols from the European Committee for Standardization (CEN prEN 14011:2002). Fish were sampled by electrofishing using a portable unit which generated up to 200 V and 3 A pulsed DC in an upstream direction. This capture method was employed as it is considered an easy and safe method, very popular for studying stream fish [2,40,41]. To minimize the potential influence of the sampling technique on the results, fish were all handled identically by the same experienced operator. 

At the start of the study (17 May 2017), several physico-chemical parameters and contaminants of emerging concern (CEC) were measured as a basis of the water quality from each sampling site (Table 1). Results of the water analyses provided evidence that the sampling sites classified as polluted presented features typically identified in disturbed environments [11,42,43].

To avoid the circadian rhythm being a source of variability, samplings were performed in the morning (10:00–12:00) from 17 May to 15 June 2017, once the constructed wetland system had been operative for 3 months (23 May, P1; 17 May, P2; 15 June, reference site). Unequal sample size was collected within the polluted sites probably due to sub-optimal habitat conditions in P1 (n = 6) compared to P2 (n = 17) or the reference site (n = 22). All procedures followed the national and institutional regulations of the Spanish Council for Scientific Research (CSIC) and the European Directive 2010/63/EU.

### 2.2. Sample Collection 

Fish were sampled after applying the combined stressor of capture and a brief period of confinement, given that stress-induced cortisol levels offer a considerable understanding of the overall stress response [44]. In this context, growing evidence suggests that circulating cortisol increases can be detected from as short as 1–2.5 min following exposure to stressors [1,21]. Accordingly, fish were caught using the portable electrofishing unit, and confined in buckets of 20 L for approximately 15 min. Afterwards, specimens were anesthetized with MS-222 (100 mg/L) and immediately after, blood and skin mucus were sampled. A heparinized insulin syringe was used to collect approximately 0.2 mL of blood by caudal vein puncture. A drop of blood was smeared for hematological analyses, and the remaining fluid kept on ice until transported back to the laboratory. Samples were then centrifuged (1500× *g*, 5 min) and plasma was collected and stored at −20 °C. Skin mucus was collected following the method described by Schultz and colleagues [45] with some modifications. Briefly, a polyurethane sponge was used to absorb the skin mucus by applying light pressure to the left and right flank as this method has been shown to be less stressful than using a spatula [45]. The sponge was then introduced into a cylinder of a syringe and compressed with the barrel to collect, into a centrifuge tube, the skin mucus. Afterwards, samples were centrifuged (2000× *g*, 10 min) and the supernatant was stored at −20 °C until analysis. Morphological variables, including length (mean ± SD: P1 = 208.5 ± 34.2 mm; P2 = 211.6 ± 22.8 mm; reference = 159.9 ± 35.5 mm) and weight (mean ± SD: P1 = 126.7 ± 61.1 g; P2 = 117.0 ± 50.0 g; reference = 58.7 ± 42.2 g) were measured. Fulton’s body condition factor (*K*), calculated according to the formula *K* = 100,000 body weight (g) total length (mm)^−3^ [46], was assessed, since it can reflect the energetic state of individuals [47]. Fish were released into the corresponding capture site once the samples had been collected. 

### 2.3. Hematological Analysis

Immediately after being collected, a drop of blood was placed on glass microscope slides, drawn across the surface and, once air-dried, slides were fixed in absolute methanol for 10 min. This procedure was run in duplicate for each specimen. Upon arrival in the laboratory, one of each duplicated slides was stained with Diff-Quick^®^ to assess the frequency of abnormal RBCs and for the WBC count. RBCs (1000) of each individual slide were scored to calculate the frequency of ENA, SE, and IE. The ENAs analyzed were defined as lobed, kidney-shaped, fragmented, and vacuolated nuclei following the directions of Pacheco and Santos [48]. The relative count of all types of WBCs (neutrophils, lymphocytes, monocytes, eosinophils, and basophils) was performed for 100 WBCs following the directions of Tavares-Dias [49]. The neutrophil and lymphocyte count was used to calculate the relative proportion of neutrophils to lymphocytes (hereafter, N:L ratio). The second duplicated slide of each individual was used to assess the number of micronucleus after performing an acridine orange staining. RBCs (3000) of each slide were scored to calculate the frequency of MN.

### 2.4. Cortisol Extraction and Biochemical Validation 

To analyze cortisol levels from blood and skin mucus, a commercial enzyme immunoassay (EIA) kit (Cortisol ELISA KIT; Neogen^®^ Corporation, Ayr, UK) was used. 

Biochemical validation of the EIA was carried out following the methods described by Carbajal and collaborators [50]. Samples of plasma and skin mucus extracts from several individuals were first pooled and used in each validation test. Precision was evaluated with the intra-assay coefficient of variation (CV), calculated from all duplicated samples analyzed. All samples from each matrix evaluated were analyzed in single assays; therefore, the inter-assay CV was not assessed. The dilution test was applied to assess the specificity of the EIA kit by comparing observed and theoretical values from pools diluted with EIA buffer. To test the assay’s accuracy, the spike-and-recovery test was used, where known volumes of pools were mixed with different volumes and concentrations of pure standard cortisol solution. Finally, we evaluated the sensitivity of the test, given by the smallest amount of cortisol concentration detected. 

### 2.5. Statistical Analysis 

The computer program R software (R-project, Version 3.0.1, R Development Core Team, University of Auckland, New Zealand) was used to analyze the data. A *p* < 0.05 was considered statistically significant. Normality of the data was assessed using Shapiro–Wilk tests, and parametric or non-parametric tests were applied accordingly. Differences in cortisol levels and hematological data between sites were assessed using one-way ANOVA with Tukey’s pairwise post-hoc tests. Non-normally distributed data were assessed by using Kruskal–Wallis test, followed by a multiple comparison test. Length and *K* were run as covariates in the models to account for potential differences across sites due to the age of the fish or the energy accumulated in the body, respectively. These covariates were then removed when results showed no influence on the response variable (*p* > 0.05). Pearson and Spearman correlation tests were applied to test for correlations between skin mucus cortisol levels to levels of the hormone in blood and the hematological variables. 

## 3. Results

In total, 45 Catalan chub from sites P1 (mean *K* ± SD = 1.17 ± 0.14, N = 6), P2 (mean *K* ± SD = 1.30 ± 0.07, N = 17), and reference (mean *K* ± SD = 1.18 ± 0.08, N = 22) were captured, sampled, and returned to the river immediately after sampling. 

### 3.1. Biochemical Validation of the EIA 

Plasma and skin mucus intra-assay CV was 8.8% and 7.7%, respectively. The dilution test obtained for plasma showed an R^2^ = 98.4% and a mean percentage error of 104.1 ± 4.1%. In the skin mucus dilution test, an R^2^ = 99.7% and a mean percentage error of 108.7 ± 8.7% was obtained. Also, in the dilution test, obtained and theoretical concentrations of plasma and skin mucus extracts showed significant correlation coefficients (*r* = 0.99, *p* < 0.01). In the spike-and-recovery test, the average of the recovery percentage was 107.6 ± 10.0% for plasma and 109.6 ± 9.1% for skin mucus. The sensitivity of the assay for plasma and skin mucus assessment was 0.07 ng cortisol/mL and 0.03 ng cortisol/mL, respectively. The biochemical validation of the EIA showed reliable results that demonstrated the assay’s precision, specificity, accuracy and sensitivity in measuring plasma and skin mucus cortisol levels of the Catalan chub.

### 3.2. Hematological Parameters and Cortisol Levels

**Figure 2:**
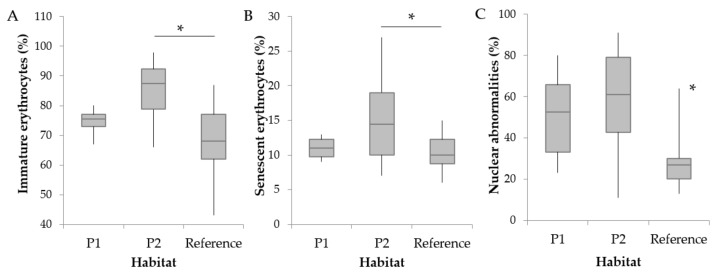
Frequencies of (**A**) immature erythrocytes, (**B**) senescent erythrocytes, and (**C**) erythrocytic nuclear abnormalities determined in the Catalan chub sampled at two polluted sites (P1, n = 6; P2, n = 17) and a reference non-impacted upstream site (n = 22) in the Besòs River. Asterisks (*) indicate significant differences between habitats (*p* < 0.05).

Concerning the RBC alterations, the frequency of IE (Figure 2A) and SE (Figure 2B) was significantly lower in the reference site compared to P2 (*p* < 0.05). In addition, significantly lower frequencies of ENA (Figure 2C) were detected in the reference site compared to both polluted habitats (*p* < 0.05). Despite no differences detected between polluted and reference sites in the frequency of MN (*p* > 0.05), a higher frequency of MN was detected in P1 compared to P2 (*p* < 0.05).

When accounting for differences in WBC counts between habitats (Table 2), we detected a higher N:L ratio in P1 and P2 compared to the reference site (*p* < 0.05), and a significant interaction effect of site with length on the N:L ratio (*p* < 0.05). Although no differences were detected between habitats in the proportion of monocytes and eosinophils (*p* > 0.05), a higher proportion of basophils was detected in P2 compared to the reference site (*p* < 0.05). 

**Figure 3:**
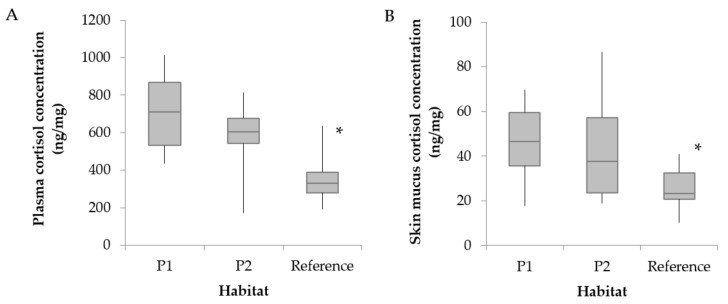
Cortisol concentrations in (**A**) plasma and (**B**) skin mucus of the Catalan chub sampled at two polluted sites (P1, n = 6; P2, n = 17) and a reference non-impacted upstream site (n = 22) in the Besòs River. Asterisks (*) indicate statistically significant differences between habitats (*p* < 0.05).

Plasma cortisol concentrations (PCC) (Figure 3A) and mucus cortisol concentrations (MCC) (Figure 3B) were significantly lower in the reference site than in both polluted sites (*p* < 0.05).

When studying the relationships between MCC, PCC, and the hematological parameters (Table 3), significant correlations were identified between MCC and PCC (*p* < 0.01), IE (*p* < 0.05), ENA (*p* < 0.05), and the N:L ratio (*p* = 0.05). 

## 4. Discussion

In this study, we first successfully validated that several physiological endpoints typically used as indicators of exposure to pollutants (abnormal RBCs and altered WBC counts), were accurately related to the habitat quality in the Catalan chub. Cortisol levels in blood were also compared between habitats and they were correlated to skin mucus cortisol concentrations. Finally, we contrasted the patterns of response of all the endpoints assessed to skin mucus cortisol levels across the sites. Lack of an adequate number of samples in one of the polluted sites (P1, n = 6) makes the cross-site comparison difficult. The discussion is therefore focused on differences detected between sites with a larger sample size (P2, n = 17; reference, n = 22). Results highlight the potential of this non-invasive tool to assess habitat quality and the need to combine regular techniques for biomonitoring the wetlands systems’ performances with the measurement of cortisol in skin mucus.

### 4.1. Abnormal RBC Frequencies 

There was no consistent pattern in abnormal RBC frequencies when they were compared between the polluted and the reference habitats. As confirmed by the physico-chemical and CEC analysis (Table 1), the site P1, located at the beginning of the wetlands system, presented slightly worse habitat conditions compared to the site P2, placed at the end of the same system. Accordingly, we expected to identify further RBC alterations in P1 than in P2. Nevertheless, relative to the reference site, P1 only exhibited significantly higher frequencies of ENA, while fish from P2 presented greater IE, SE, and ENA frequencies. As mentioned earlier, the sub-optimal conditions of P1 were likely the cause of the small sample size collected in the site, which in turn could have limited the statistical power in detecting potential differences.

Conversely, both polluted P1 and P2 sites showed clearly higher ENA levels than the reference habitat. Particularly in spring, greater frequencies of nuclear abnormalities in the same fish species have already been identified [11]. The ENA test has been demonstrated to be a highly sensitive parameter for pollution assessment [12,51], probably explaining the clear variations detected in these nuclear abnormalities between habitats. 

Besides the inconsistencies found between both polluted sites, higher frequencies of RBC disorders detected in the polluted habitat with larger sample size further support the idea that IE, SE, and ENA tests are reliable biomarkers of habitat quality in the Catalan chub [52,53]. 

Although these commonly noted abnormalities are highly sensitive to pollution, they are not as widely accepted as the use of MN tests [53,54]. The influence of river status on the frequency of MN has been previously studied in the Catalan chub, and, in accordance with our results, no changes were detected between degraded and reference streams [11]. Nevertheless, in our study, the two polluted areas evaluated differed in MN levels between them, with the highest values detected at the end of the wetland system. Although this result could also be given by the different sample size between study sites, it should be noted that a different contaminant profile can also result in mismatch between habitats [13]. For example, exposure to atrazine and ametrine herbicides resulted in increased MN [55]; in contrast, a different herbicide, pendimethalin, showed increased ENA but not MN [56]. Tetrachloroethene, the only CEC analyzed that presented higher concentrations in P2 than P1, is a dry-cleaning compound widely used in the textile industry, known to have toxic effects in fish [57,58]. Although evidence in humans supports the link between the MN formation and this compound [59], to the authors’ knowledge, there are no published studies demonstrating this association in fish. Despite this, other compounds not specifically analyzed in this study could also be the consequence of the differing results between polluted habitats. 

Taken together, these findings suggest that the detection of RBC disorders can be potentially used to identify low-quality habitats for the Catalan chub, while being an approach that could contribute to a better understanding of the species’ health status than the MN test.

### 4.2. Variations in WBC Counts 

Characteristic changes in blood leukocyte counts have been generally linked to the continuous activation of the HPI axis [2,60,61,62]. Interestingly, prolonged exposure to environmental contaminants can cause neutrophilia and/or lymphopenia in fish [16,17,18], likewise in other taxa [9,63]. In line with these published reports, different WBC counts were detected between the study sites. Most notably, the N:L ratio was significantly higher in both polluted sites compared to the reference stream. These between-site differences could be a consequence of the sub-optimal environmental conditions in P1 and P1 habitats. However, the significant interaction detected between length (age) of fish and site suggests caution when interpreting results. Although length did not influence any of the other response variables tested, it remains possible that differences in the N:L ratio were partly exacerbated by the smaller size of fish sampled at the reference site. Age can be an important factor in shaping the stress response [2]; nevertheless, further studies are needed to understand the influence that age/length can have in the leukocyte profile. The number of basophils, a cell type still not assessed in this species, was also higher in P2 compared to the reference site. Although the function of this cell type is poorly understood, probably because its occurrence in teleost fish seems to be very rare [49,64], basophils have been related to acute inflammation processes [16]. Besides this, neither the monocyte nor the eosinophil count appeared to differ between sites, similar to earlier findings on silver carp (*Hypophthalmichthys molitrix*) in response to pesticides [65]. The assessment of these cell types is not common in contemporary research, perhaps due to the controversy concerning the effect of stress on eosinophil and monocyte numbers [2,16]. When only WBC data are available, evaluation of these two leukocyte types can help distinguish stress from infectious responses [16], thus further research on monocyte and eosinophil changes is strongly encouraged.

### 4.3. Changes in Cortisol Levels 

Cortisol levels detected in plasma and skin mucus within the same individuals displayed a close linear relationship, suggesting that cortisol diffuses to the skin mucus in proportion to the amount of circulating hormone. Validation of alternative matrices for HPI axis activity assessment should prove that hormone concentrations in these media are proportional to their abundance in the bloodstream [3,66], as the present study demonstrates for the measurement of cortisol in skin mucus. These results, therefore, increase the applicability of the method as a sensitive-individual measure of the HPI axis activity in wild freshwater fish within their natural environment. 

In addition, both plasma and skin mucus cortisol levels differed significantly between habitats of different quality, with the highest hormone values observed in the polluted sites assessed. This association between cortisol concentrations and habitat quality suggests that variation in the HPI axis activity is likely to be related to the presence of environmental disturbances. Greater stress responses attributed to the effects of pollutants have been reported in several fish species [22,67,68], as well as in other taxa [69,70,71]. Nevertheless, it is important to note that chronic exposure to certain aquatic contaminants can also have suppressive effects on the stress axis [72,73,74,75]. In this context, investigating the toxic mechanisms underlying variation in the HPI axis alteration will be particularly informative. 

### 4.4. Integrated Assessment

Interpreting cortisol fluctuations in free-living vertebrates is certainly a complex practice, particularly when applying alternative matrices for hormone assessment [6,9]. This is why linking cortisol levels to other endpoints of the stress responses can significantly enhance the current understanding on the ecology of stress [76]. 

In the present study, fluctuations in skin mucus cortisol levels between habitats paralleled those detected in blood, the traditional matrix used for hormone assessments in fish [77]. Relative to habitat quality, changes of the hormone in skin mucus also coincided with variations in the hematological parameters, except for MN levels. Furthermore, the amount of cortisol in skin mucus was directly proportional to frequencies of abnormal erythrocytes (IE and ENA) and to the well-established stress index N:L ratio. Red blood cells are highly sensitive to landscape disturbances [78] and, more specifically, to environmental pollution [79,80]. Accordingly, the measurement of abnormal erythrocytes has been successfully used to assess the health status of the Catalan chub [11,42] and many other fish species [12,13,17]. In the same context, WBC counts, particularly the N:L ratio, increases in individuals exposed to heavy metals and other contaminants proportional, indeed, to the circulating cortisol levels [16]. Given the very clear effect of pollution on leukocyte and erythrocyte profiles, the strong linkages detected in this study provide new evidence that the measurement of cortisol in skin mucus could be potentially used as a biomarker of habitat quality in freshwater fish residing polluted environments. 

The use of a robust sample size is recommended in natural settings where individuals are exposed to different environmental conditions [9,81]. However, capturing a relevant number of individuals in wild conditions may not always be possible, especially in highly degraded habitats such as the one included in the present field experiment. An important limitation of our study design may, therefore, be the small sample size in one of the polluted habitats, which requires results of this site be interpreted with caution.

While physical and chemical techniques are commonly applied for wetland monitoring [39], methodologies that provide information about how animals are influenced by their environment are only occasionally used. The incorporation of biomarkers into the constructed wetlands’ management would provide complementary data to the conventional analyses. Hence the demonstrated sensitivity of the methods evaluated in the present study to different pollution gradients could be exploited for biomonitoring the wetlands systems’ performances. Indeed, the non-invasive measurement of cortisol in skin mucus would largely improve our understanding about the link between the detected chemical concentrations and the biological effects observed.

## Figures and Tables

**Table 1:** Occurrence of contaminants of emerging concern (CEC) and physico-chemical data from river water samples collected within the wetland system (P1 and P2) and the reference non-impacted site located outside the system (Reference) in May 2017, when fish were sampled.

Compound	Sites		
	P1	P2	Reference
CEC (µg/L)			
Volatile organic compounds			
Tetrachloroethene	<LOD	0.6	<LOD
Pesticides			
Simazine	0.13	0.13	<LOD
Diuron	<LOD	<LOD	<LOD
Isoproturon	0.04	0.04	<LOD
Pharmaceutical products			
Diclofenac	1.61	0.29	<LOD
Alkylphenols			
4-tert-octylphenol	0.025	<LOD	<LOD
Nonylphenol	0.14	<LOD	<LOD
Physico-Chemical Data (mg/L)			
NH_4_^+^	12.6	10.6	0.07
NO_3_^−^	3.31	2.83	0.18
PO_4_^−^	0.8	1	0.4
TOC ^1^	9.25	6.39	2.16
COD ^2^	29.9	30.5	5.88
SS ^3^	7	9.5	0.5
Turbidity (NTU ^4^)	4.47	3.01	0.66

Concentrations of compounds under the instrumental detection limit (LOD, Limit of detection) are not included. ^1^ TOC, total organic carbon; ^2^ COD, chemical oxygen demand; ^3^ SS, suspended solids; ^4^ NTU, nephelometer turbidity units.

**Table 2:** Mean values and standard deviation of white blood cell parameters (‰) determined in the Catalan chub from polluted (P1, n = 6; P2, n = 17) and reference (n = 22) sites in Besòs River. Different letters indicate significant differences among sites (*p* < 0.05).

White Blood Cell Type	Sites		
	P1	P2	Reference
N:L ratio	10.54 ± 4.49 ^a^	8.00 ± 3.46 ^a^	4.79 ± 2.91 ^b^
Monocytes	4.50 ± 3.67	7.47 ± 3.83	6.05 ± 2.82
Eosinophils	0.67 ± 0.82	1.35 ± 1.17	1.18 ± 1.30
Basophils	0.33 ± 0.52 ^ab^	1.24 ± 1.20 ^a^	0.36 ± 0.58 ^b^

**Table 3:** Correlation (*r*) and *p*-value between hematological variables and skin mucus cortisol concentrations (MCC).

Variable	MCC (*r*)	*p*-Value
Cortisol		
Plasma cortisol concentration	0.55	**<0.01**
Red blood cells		
Immature erythrocytes	0.40	**0.03**
Senescent erythrocytes	0.23	0.24
Erythrocytic nuclear abnormalities	0.41	**0.02**
Micronucleus	−0.001	0.99
White blood cells		
N:L ratio	0.34	**0.05**
Monocytes	−0.01	0.97
Eosinophils	−0.21	0.20
Basophils	0.24	0.17

Bold numbers denote significant correlations between the hematological variables and MCC.
